# Incidence, Clinical Characteristics, and Survival of Collecting Duct Carcinoma of the Kidney: A Population-Based Study

**DOI:** 10.3389/fonc.2021.727222

**Published:** 2021-09-14

**Authors:** Chaopeng Tang, Yulin Zhou, Silun Ge, Xiaoming Yi, Huichen Lv, Wenquan Zhou

**Affiliations:** Department of Urology, Jinling Hospital, Jinling School of Clinical Medicine, Nanjing Medical University, Nanjing, China

**Keywords:** collecting duct carcinoma, kidney, SEER database, surgery, chemotherapy

## Abstract

**Objective:**

To investigate the exact age‐adjusted incidence (AAI), clinical characteristics, and survival data of collecting duct carcinoma of the kidney (CDCK) recorded in the Surveillance, Epidemiology, and End Results (SEER) database of the National Cancer Institute.

**Methods:**

Patients with CDCK confirmed by microscopic examination from 2004 to 2018 were selected from the SEER database. AAI rates were calculated using SEER*Stat software (version 8.3.9). The Kaplan‐Meier method was used to evaluate cancer-specific survival (CSS) rates according to tumor size, tumor stage, and treatment methods, and differences among these variables were assessed by the log‐rank test. Cox regression analysis was employed to identify variables independently related to CSS.

**Results:**

A total of 286 patients with CDCK were identified from the database. The majority of the patients were white (69.2%), male (67.5%), and married (60.5%), and the median age was 59 years. Most patients with CDCK (74.4%) presented with stages III or IV disease. The diameter of most (59.4%) tumors was less than 7 cm, and the tumors were more commonly found on the left than on the right (55.2% *vs.* 44.8%). The incidence of CDCK decreased over time. The median CSS time was 17 months. In terms of the treatment modalities used, 83.9% of the patients underwent surgery; 32.9% underwent chemotherapy, and 13.6% underwent radiotherapy. The CSS rates at 1, 2, and 5 years were 57.3%, 43.2%, and 30.7%, respectively. In patients with stage IV CDCK treated with surgery alone, chemotherapy alone, and surgery plus chemotherapy, the median survival time was 5 months, 9 months, and 14 months, respectively (*P* =0.024). Multivariate Cox regression analysis showed surgery, chemotherapy, stage, regional lymph node metastasis, and distant metastasis were independent prognostic factors for patients with CDCK.

**Conclusions:**

CDCK is an uncommon malignant renal carcinoma, and its incidence is decreasing based on the analysis of current data. CDCK is a high stage, regional lymph-nodes positive, and metastatic disease. Compared with surgery alone or chemotherapy alone, patients with stage IV could gain survival benefit from surgery combined with chemotherapy.

## Introduction

Collecting duct carcinoma of the kidney (CDCK), which is believed to arise from the epithelial layer of the collecting ducts of Bellini in the renal medullary, is an uncommon pathological subtype of renal carcinoma and accounts for 0%–3% of all renal malignancies (1–3). According to histologic findings, CDCK is defined as a subtype of renal carcinoma. However, the presentation, imaging findings, and prognosis of CDCK remarkably differ from those of other types of renal cancer. Clinically, CDCK displays characteristics similar to those of upper tract urothelial cell carcinoma (UTUC), which has a poor prognosis and limited response to immunotherapy (3, 4).

No significant differences were observed in terms of the effect of race or sex on the relative survival rates of CDCK in 98 samples recorded from 1973 to 2004 in the Surveillance, Epidemiology, and End Results (SEER) database (5). Wright JL et al. (6) compared cases of CDCK and clear cell renal cell carcinomas (CCRCC) recorded in the SEER database from 2001 to 2005 and found that patients with CDCK had a higher stage and poorer prognosis compared with CCRCC. This result is consistent with the largest report on CDCK by Sui W et al. (7). Abern MR et al. (8) compared the cancer-specific survival (CSS) rates of medullary renal cell carcinoma and CDCK from 1995 to 2007 recorded in the SEER database, and found that the prognosis of both diseases is generally poor; moreover, locally high stage or metastatic disease and not receiving surgery were considered predictors of mortality in the CDCK model.

Although these studies based on the SEER database clarified the clinical characteristics and prognosis of patients with CDCK, they did not investigate the association of variables such as marital status, radiotherapy, and chemotherapy with survival. Considering the rarity of this disease, we analyzed the latest CDCK data in the SEER database from 2004 to 2018 to identify the age‐adjusted incidence (AAI) rates, clinical features, independent predictors of CSS rates, and survival outcomes of CDCK.

## Materials and Methods

### Study Population

We used the SEER database, which included 18 tumor registries released in April 2021, for analysis. The SEER program collected cancer data from population-based registries representing about 34.6% of the US population. The database was a public and free research resource.

Patients with CDCK were selected based on the code of ICD-0-3: 8319/3: Collecting duct carcinoma. The eligibility criteria were as follows: (a) the labeled tumor sequence number was “one primary only;” (b) the year of diagnosis was between 2004 and 2018; (c) the labeled primary site was selected as “C64.9-Kidney, NOS;” (d) the tumor was microscopically confirmed; and (e) the tumor was unilateral. Patients with an unknown survival time were excluded from this work. The final cohort included 286 CDCK patients who met the eligibility criteria. Flowchart displaying the selection procedure of CDCK cases in the SEER database is shown in **Supplementary Figure 1**.

### Patient Information

The “case listing session” option was used to obtain the information of demographic factors such as age at diagnosis, sex, race, and marital status at diagnosis. Tumor features, including tumor size, tumor site, stage, T, N, and M were also extracted from the database. Furthermore, we collected information on treatment methods, including surgery, radiotherapy, and chemotherapy.

### Statistical Analysis

The AAI rates of CDCK were calculated using SEER*Stat software (version 8.3.9). The incidence rates were adjusted to the 2000 US population. The X-tile software determined 72 and 7 as the optimal cutoff point for age and tumor diameter, respectively. Demographic and clinical factors were analyzed using descriptive statistics, and the chi-square test was used to assess differences among annual incidence rates. Median, 1-year, 2-year, and 5-year overall survival (OS) and CSS rates were estimated using the Kaplan–Meier method. Kaplan–Meier curves were used to assess the CSS rates according to demographics, clinical parameters, and treatment methods, and the log‐rank test was used to assess differences among these variables, the analysis was further stratified by tumor stage that could affect the treatment effects. Cox regression analysis was employed to identify variables independently related to CSS. SPSS version 21 was used for statistical analyses, and *P*<0.05 was considered significant.

## Results

### Incidence of CDCK

**Figure 1:**
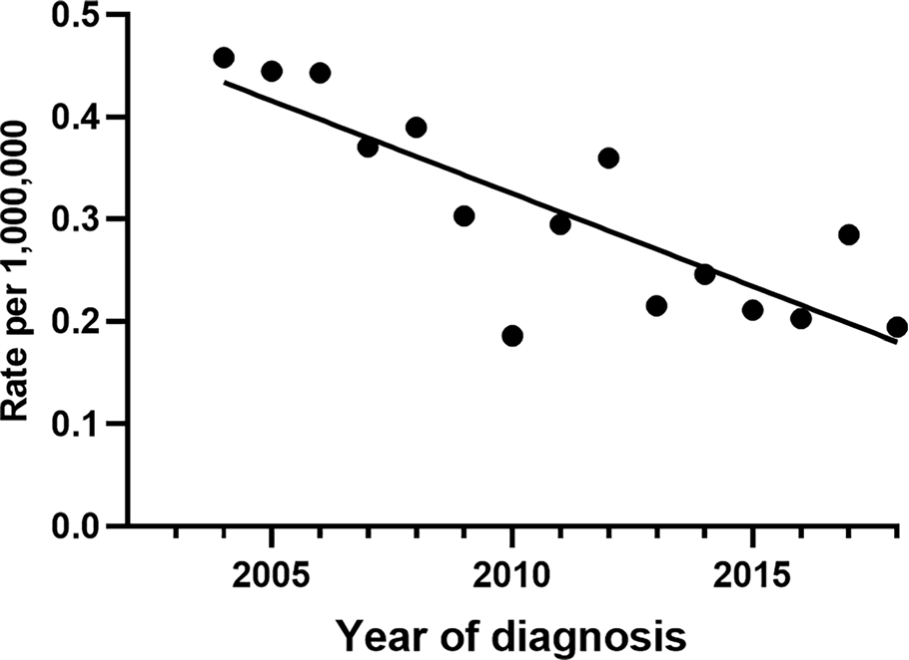
Incidence of CDCK in the period 2004–2018.

The overall AAI of CDCK between 2004 and 2018was 0.2990 per 1,000,000 population. **Figure 1** shows that the incidence rate of CDCK decreased annually from 0.4580 per 1,000,000 population in 2004 to 0.1943 per 1,000,000 population in 2018. A significant difference in incidence rates was observed between these years (*P* =0.002). The AAI of CDCK showed a decreasing trend over time.

### Clinical Characteristics of CDCK

The demographic characteristics of the patients showed that the median age in this study was 59 years (range: 14–89 years). In terms of marital status, 16.8% of the patients were single, 60.5% were married, and 22.7% were classified as others (including divorced, widowed, unknown, and others). In terms of sex, male patients accounted for 67.5% of the study population, and the male-to-female ratio was 2.08:1. The majority of the patients (69.2%) were white, 21.3% were black, and 9.4% were identified as others (including American Indian/Alaska Native, Asian or Pacific Islander, and unknown). In terms of tumor characteristics, 18.9%, 4.2%, 24.1%, and 50.3% of the patients presented with stages I, II, III, and IV disease, respectively. The proportions of regional lymph nodes and distant metastasis were 40.6% and 42.0%, respectively. Most patients (59.4%) had tumors less than 7 cm in diameter, and the diameter range was 1.2–24.8 cm. Tumors were more commonly located on the left side than on the right side of the body (55.2% *vs.* 44.8%). With regard to the treatment modalities used, surgery was the primary therapy (83.9%) for CDCK patients. Only a few patients received radiotherapy or chemotherapy, and the ratios of patients receiving these treatments relative to the total study population were 13.6% and 32.9%, respectively. Furthermore, beam radiation was the main radiotherapy scheme. The relevant demographic and clinical characteristics of the patients with CDCK are shown in **Table 1**.

**Table 1:** Descriptive data of patients with CDCK.

Variables	Number (%)
Marital status	
Married	173 (60.5%)
Single	48 (16.8%)
Others	65 (22.7%)
Race	
White	198 (69.2%)
Black	61 (21.3%)
Others	27 (9.4%)
Sex	
Male	193 (67.5%)
Female	93 (32.5%)
Age (years)	
≤72	233 (81.5%)
>72	53 (18.5%)
Tumor site	
Left	158 (55.2%)
Right	128 (44.8%)
Surgery	
Yes	240 (83.9%)
No	46 (16.1%)
Radiotherapy	
Yes	39 (13.6%)
No/Unknown	247 (86.4%)
Chemotherapy	
Yes	94 (32.9%)
No/Unknown	192 (67.1%)
Tumor size	
≤7 cm	170 (59.4%)
>7 cm	102 (35.7%)
Unknown	14 (4.9%)
Tumor stage	
I	54 (18.9%)
II	12 (4.2%)
III	69 (24.1%)
IV	144 (50.3%)
Unknown	7 (2.4%)
T	
T1	79 (27.6%)
T2	18 (6.3%)
T3	151 (52.8%)
T4	26 (9.1%)
Tx	12 (4.2%)
Regional lymph node metastasis	
N0	158 (55.2%)
N1	116 (40.6%)
Nx	12 (4.2%)
Distant metastasis	
M0	162 (56.6%)
M1	120 (42.0%)
Mx	4 (1.4%)

### Prognosis of CDCK

**Figure 2:**
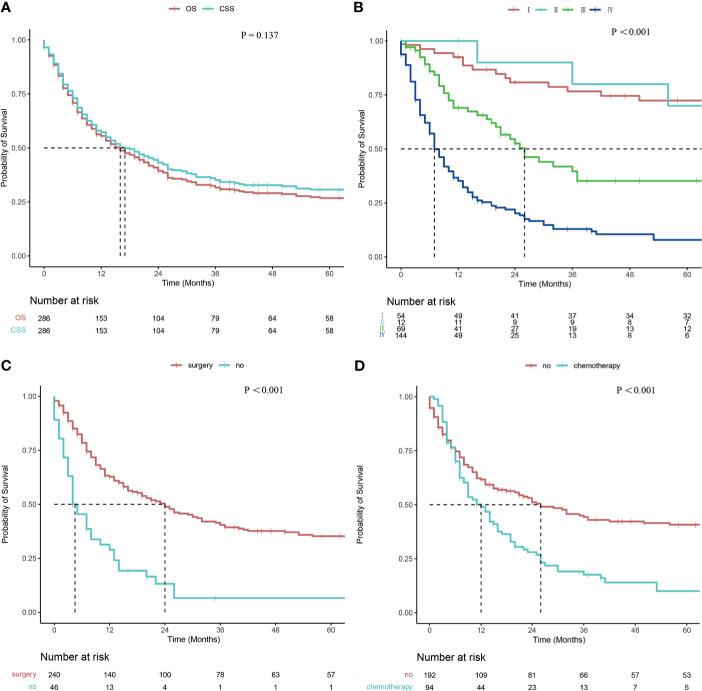
Survival curve of patients with CDCK evaluated using the Kaplan–Meier method. **(A)** OS and CSS; **(B)** According to the stage; **(C)** According to surgery; **(D)** According to chemotherapy.

The mean follow-up period was 33.6 months (range 0-175 months). The median OS and CSS times were 16 months (95% CI: 11.718–20.282) and 17 months (95% CI: 11.850–22.150), respectively. The OS rates at 1, 2, and 5 years were 55.5%, 39.4%, and 26.8%, respectively. The CSS rates at 1, 2, and 5 years were 57.3%, 43.2%, and 30.7%, respectively. No significant difference was observed between OS and CSS (*P* =0.137, **Figure 2A**).

Patients with smaller tumors have higher survival rates than those with larger tumors (24 months *vs.* 13 months, *P* = 0.038). The survival rates were also related to T staging, and the median survival times of T1, T2, T3, and T4 were 81 months, 56 months, 13 months, and 7 months, respectively (*P <*0.001). A statistical difference was observed between T1 and T3 disease (*P <*0.001), between T1 and T4 disease (*P <*0.001), between T2 and T3 disease (*P* =0.022), between T2 and T4 disease (*P* =0.001), and between T3 and T4 disease (*P* =0.040), but not between T1 and T2 disease (*P* =0.813**)**. The median survival times of patients with and without regional lymph node metastases were 9 and 41 months, respectively (*P <*0.001**)**. Differences in median survival times between patients with and without distant metastases were statistically significant at 7 and 53 months, respectively (*P <*0.001**)**. The survival rates were related to tumor stage, and the median survival times for stages I, II, III, and IV were as follows: not reached, 91 months, 26 months, and 7 months, respectively (*P <*0.001; **Figure 2B**). Pairwise comparison indicated a statistical difference between stage I and stage III disease (*P <*0.001), between stage I and IV disease (*P <*0.001), between stage II and III disease (*P* =0.049), between stage II and IV disease (*P <*0.001), and between stage III and IV disease (*P <*0.001), but not between stage I and stage II disease (*P* =0.706**)**. The CSS rates were significantly higher in surgery patients than non-surgery patients (24 months *vs.* 4 months, *P <*0.001; **Figure 2C** **)**. A significant difference in CSS rates was also observed between radiotherapy patients and non-radiotherapy patients (8 months *vs.* 23 months, *P <*0.001**)**. Patients treated with chemotherapy had lower CSS rates than those without chemotherapy (12 months *vs.* 26 months, *P <*0.001; **Figure 2D** **)**. Univariate analysis revealed no significant difference in survival rates with regard to marital status, sex, race, age, and tumor site.

**Figure 3:**
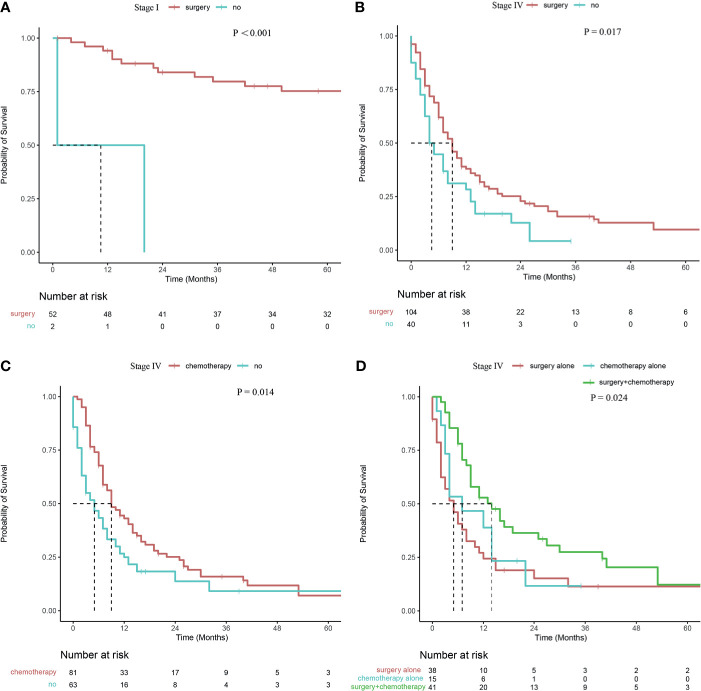
Survival curve of patients with CDCK evaluated using the Kaplan–Meier method **(A, B)** Surgery stratified according to tumor stage; **(C)** Chemotherapy stratified according to tumor stage; **(D)** Surgery and/or chemotherapy alone stratified according to tumor stage.

In terms of radiotherapy stratified according to tumor stage, no significant difference was observed in survival rates between patients with stage III or IV (*P* =0.107, *P* =0.520, respectively). In terms of surgery stratified according to tumor stage, patients presenting with stage I or IV CDCK who underwent surgery had higher survival rates than those patients without surgery (*P <*0.001, and *P* =0.017, respectively; **Figures 3A, B**). However, no significant differences were observed in patients presenting with stage III disease (*P* =0.715), and all patients presenting with stage II CDCK underwent surgery. In terms of chemotherapy stratified according to tumor stage, patients presenting with stage IV CDCK who underwent chemotherapy had higher survival rates than patients without chemotherapy (*P* =0.014; **Figure 3C**). However, no significant differences were observed in patients presenting with stages II and III (*P* =0.069, and *P* =0.779, respectively). All patients who presented with stage I disease did not undergo chemotherapy. Patients presenting with stage IV CDCK who underwent surgery plus chemotherapy had higher survival rates than those who underwent surgery or chemotherapy alone (14 months, 5 months, and 9 months, respectively; *P* = 0.024; **Figure 3D** **),** while survival rates were similar between patients who underwent surgery or chemotherapy alone (*P* = 0.505). However, there were no significant differences in survival rates between patients with stage III treated with surgery plus chemotherapy or surgery alone (23 months *vs.* 29 months; *P* = 0.850).

Variables exerting significant differences in survival rates were enrolled in the multivariate Cox regression analysis. The results of this analysis are shown in **Table 2**. Univariate Cox regression analysis suggested that no surgery (HR: 2.983; 95%CI: 2.080–4.277; *P <*0.001), radiotherapy (HR: 2.291; 95%CI: 1.579–3.322; *P <*0.001), chemotherapy (HR: 1.805; 95%CI: 1.339–2.432; *P <*0.001), tumor size >7 cm (HR: 1.429; 95%CI: 1.055–1.937; *P*=0.021), regional lymph node metastasis (HR: 2.724; 95%CI: 2.004–3.701; *P <*0.001), stage III(HR:2.953; 95%CI 1.636–5.331; *P <*0.001), stage IV(HR: 7.514; 95%CI 4.407–12.813; *P <*0.001), T3(HR: 2.268; 95%CI 1.557–3.303; *P <*0.001), T4(HR: 3.599; 95%CI 2.132–6.074; *P <*0.001), and distant metastasis (HR: 4.204; 95%CI: 3.084–5.730; *P <*0.001) were risk factors for prognosis. Finally, multivariate Cox analysis revealed that surgery, chemotherapy, stage, regional lymph node metastasis, and distant metastasis were independent prognostic factors for CDCK patients.

**Table 2:** Univariate and multivariate analyses of the CSS of the patients.

	Univariate		Multivariate	
Variables	HR (95%CI)	*P* value	HR (95%CI)	*P* value
Marital status				
Married	1			
Single	0.840 (0.562–1.254)	0.394		
Others	0.807 (0.553–1.178)	0.267		
Race				
White	1			
Black	0.752 (0.518–1.092)	0.134		
Others	0.819 (0.487–1.377)	0.452		
Sex				
Male	1			
Female	0.803 (.586–1.100)	0.172		
Age				
≤72	1			
>72	1.091 (0.741–1.606)	0.660		
Tumor site				
Left	1			
Right	1.151 (0.861–1.540)	0.342		
Surgery				
Yes	1		1	
No	2.983 (2.080–4.277)	<0.001	1.814 (1.126–2.924)	0.014
Radiotherapy				
No/Unknown	1		1	
Yes	2.291 (1.579–3.322)	<0.001	1.184 (0.800–1.753)	0.398
Chemotherapy				
No/Unknown	1		1	
Yes	1.805 (1.339–2.432)	<0.001	0.454 (.312–.661)	<0.001
Tumor size				
≤7 cm	1		1	
>7 cm	1.429 (1.055–1.937)	0.021	1.153 (0.820–1.622)	0.412
Unknown	1.585 (0.828–3.037)	0.165	0.958 (0.401–2.293)	0.924
Tumor stage				
I	1		1	
II	1.168 (0.390–3.494)	0.782	0.793 (0.180–3.503)	0.760
III	2.953 (1.636–5.331)	<0.001	2.218 (0.997–4.934)	0.051
IV	7.514 (4.407–12.813)	<0.001	2.565 (1.055–6.236)	0.038
Unknown	4.133 (1.377–12.401)	0.011	2.340 (0.386–14.173)	0.355
T				
T1	1		1	
T2	1.081 (0.522–2.235)	0.834	1.371 (0.486–3.870)	0.551
T3	2.268 (1.557–3.303)	<0.001	1.381 (0.775–2.461)	0.274
T4	3.599 (2.132–6.074)	<0.001	1.566 (0.828–2.962)	0.168
Tx	2.002 (0.892–4.493)	0.092	0.467 (0.136–1.605)	0.226
Regional lymph node metastasis				
N0	1		1	
N1	2.724 (2.004–3.701)	<0.001	1.448 (1.001–2.096)	0.049
Nx	2.478 (1.192–5.150)	0.015	2.268 (0.773–6.649)	0.136
Distant metastasis				
M0	1			
M1	4.204 (3.084–5.730)	<0.001	3.256 (1.844–5.750)	<0.001
Mx	1.686 (0.414–6.867)	0.466	1.346 (0.173–10.479)	0.777

## Discussion

CDCK, also known as Bellini duct carcinoma, is a relatively uncommon and aggressive malignant tumor originating from the epithelial layer of the collecting ducts of Bellini in the renal medullary (9). Studies suggest that patients with CDCK are associated with a high incidence of early mortality; specifically, 60% –70% of patients died within 3 years of diagnosis (10). The first observation that Bellini duct epithelial cells are the source of tumor development was reported by Cromie W et al. (11). However, Fleming and Lewi identified the disease as a unique renal cell carcinoma in 1986 (12). Our literature search revealed that studies on CDCK are gradually increasing. However, the specific incidence and AAI of CDCK remain unclear; indeed, the literature reports a CDCK incidence rate of only 0%–2% of all renal malignancies (1, 2). Our results showed that the overall AAI of CDCK between 2004 and 2018 was 0.299 per 1,000,000 population, and numbers showed a decreasing trend annually.

In a cohort of 227 CDCK cases reported from 1995 to 2007 in the SEER database, the median CSS time was 30 months. However, in another cohort of 160 CDCK cases from 2001 to 2005 in the SEER database, the median survival time was only 5 months (6). Our study showed that the median CSS and OS of 286 CDCK patients were 17 and 16 months, respectively; these results are consistent with the largest known cohort regarding CDCK (7). A previous study based on the SEER dataset reported that the 1- and 3-year CSS rates for CDCK were 70% and 58%, respectively, and we reported that the OS rates at 1, 2, and 5 years were 55.5%, 39.4%, and 26.8%, respectively. Moreover, the CSS rates at 1, 2, and 5 years were 57.3%, 43.2%, and 30.7%, respectively, consistent with the findings of May et al. (i.e.,60.4%, 47.3%, and 40.3%, respectively) (13), thus indicating that over 50% of the CDCK patients died within 2 years. Inconsistences between the results recorded in the present work and previous reports based on the SEER dataset may be due to differences in the inclusion criteria among studies.

In our study, the ages of the patients ranged from 14 years to 89 years, with a median age of 59 years. Males were more likely to develop CDCK than females, and the ratio of males to females was 2.08:1. This result is consistent with previous reports (8, 14), that is, CDCK is frequently found in middle-aged and older patients and more often observed in men than in women. Moreover, tumors occurring on the left side have a slight advantage over those on the right side.

Hematuria is the most common presentation of CDCK, followed by abdominal pain, weight loss, and palpable masses. The clinical symptoms of CDCK vary according to tumor size, location, and invasion. Some patients are diagnosed by physical examination because they have no symptoms (15), whereas other patients are diagnosed with symptoms at the site of metastasis (16, 17). Unfortunately, the absence of information on symptoms in the database prevented us from understanding the characteristics of patients’ symptoms in the present study.

The preoperative diagnosis of CDCK is limited by the lack of specific radiological features. When the tumor is small, the imaging features supporting the diagnosis of CDCK include solitary tumor, medullary location, weak and heterogeneous enhancement, renal sinus involvement, infiltrative growth, and continuous renal contour (9, 18). However, when the tumor is large, the expansive growth of the tumor obscures these features; thus, distinguishing CDCK from other common cortical renal cell carcinomas is difficult (19). Studies (20, 21) found that CDCK showed high 18-fluorine fluorodeoxyglucose (^18^F-FDG) uptake in local and distant metastases; therefore, ^18^F-FDG-PET/CT may be an appropriate method to assess the extent of the disease.

Given the lack of specific imaging findings, distinguishing CDCK from other tumors by imaging alone is extremely challenging. Therefore, the correct diagnosis still depends on pathological examination. The International Society of Urological Pathology believes that a tumor should present with the following pathological features to be diagnosed as CDCK: (1) tumor involving the renal medullary, (2) predominant tubule formation, (3) present with desmoplastic stromal reaction, (4) cytologic features are high grade, (5) infiltrative growth pattern, and (6) other RCC subtypes of urothelial carcinoma should be excluded (22).

CDCK needs to be differentiated from urothelial carcinoma, renal medullary carcinoma, papillary renal cell carcinoma type 2, and unclassified renal cell carcinoma (23). Differentiating CDCK from UTUC is especially critical. Immunohistochemistry can be used to determine the origin of the tumors based on the staining characteristics of each cell type for differential diagnosis. A number of markers suggest that CDCK expresses some specific biological markers, including UEA-1, PNA, HMW-CK, and Fez1 (24). Collecting ducts express PAX8, and p63 is a marker that is commonly used for urothelial differentiation. Albadine R et al. (25) found that the combination of PAX8 and p63 can accurately differentiate between CDCK and UTUC. The diagnosis of CDCK was supported when PAX8+/p63−, the sensitivity and specificity were 85.7% and 100%, respectively; by contrast, the diagnosis of UTUC was supported when PAX8−/p63+, the sensitivity and specificity were 88.2% and 100%, respectively.

The majority of patients with CDCK present with highly metastatic features and an advanced TNM stage at diagnosis (15). The lymph nodes, lungs, liver, bones, and adrenal glands are the most frequent distant metastases sites (26). In this study, regional lymph node metastasis occurred in 116 out of 286 cases, and distant metastasis occurred in 120 out of 286 cases. We found that CSS rates decreased with increasing tumor stage. Our finding is consistent with the results of previous studies (7), which showed that advanced-stage disease is an independent predictor for poor survival. Our study showed that the hazard ratios of survival rates in patients with CDCK of stages II, III, and IV were 1.168, 2.953, and 7.514 times higher than that in patients with stage I CDCK, respectively. Compared with that of T1, the hazard ratios of survival rates in T2, T3, and T4 were 1.081, 2.268, and 3.599, respectively. The hazard ratio of the survival rates of patients with lymph node metastases was 2.724 times higher than that of patients without lymph node metastases. The hazard ratio for the survival of patients with metastases was 4.204 higher compared with that of patients without distant metastases. Multivariate Cox regression analysis showed that stage, regional lymph node metastasis, and distant metastasis were independent prognostic factors for CDCK patients.

Although limited reports established that targeted therapy and immunotherapy could be beneficial for patients with advanced CDCK (27–30), the prognosis of patients with CDCK remains poor, cytoreductive nephrectomy may be the only potentially curable option for patients with CDCK (8, 27, 31). The majority of the reported patients were treated with radical nephrectomy, while a few cases were treated with partial nephrectomies (32, 33). Given the aggressive nature of CDCK, radical nephrectomy is usually recommended (15), partial nephrectomy may be a treatment option in the management of low-grade CDCK (32). In our study, surgery, including cryosurgery, nephrectomy, and ureterectomy, was the first option. Of 286 patients, 240 received surgery. The median survival times of surgery and non-surgery patients were 24 and 4 months, respectively, and a significant difference in CSS rate was observed between these patients. Considering that CDCK is prone to lymph node metastasis, lymph node metastasis was found in 40.6% of the patients in this study. Therefore, the hilar lymph node should be removed during surgery.

Literature related to CDCK chemotherapy is limited. Because of the similarity of the clinical features of CDCK and urothelial cancer, some researchers believe that the chemotherapy regimen for urothelial cancer may be effective for patients with CDCK. Milowsky MI et al. (34) used doxorubicin and gemcitabine for postoperative chemotherapy in a CDCK patient. Although early treatment effects were favorable, the patient died 10 months after a diagnosis of bone and liver metastasis. Peyromaure M et al. (35) reported two CDCK patients who received postoperative chemotherapy, including gemcitabine and cisplatin, and remained disease-free for 27 and 9 months postoperatively. In the present study, however, the median survival time of patients who underwent chemotherapy was significantly lower than that of patients without chemotherapy. The inconsistent results compared with other reports may be contributed to differences in composition between the two groups, that is, 1, 12, and 81 out of 94 patients with chemotherapy had stage II, III, and IV tumors, respectively; by contrast, 54, 11, 57, and 63 out of 192 patients without chemotherapy had I, II, III, and IV tumors, respectively. Stratified analysis was performed to exclude the interference of the confounding factors on the treatment effect. In terms of chemotherapy stratified according to tumor stage, patients with stage IV CDCK who underwent chemotherapy had higher survival rates than patients without chemotherapy. However, no significant differences were found among patients with stages I, II, and III. In terms of surgery stratified according to tumor stage, patients presenting with stage I or IV CDCK who underwent surgery had higher survival rates than those patients without surgery. However, no significant differences were observed in patients presenting with stage III disease. Patients presenting with stage IV CDCK who underwent surgery plus chemotherapy had higher survival rates than those who underwent surgery or chemotherapy alone, these results are consistent with the previous literature (7). Considering that CDCK has genetic characteristics that differ from those of UTUC, chemotherapy regimens used for uroepithelial carcinoma are unsuitable for the former (36), and new regimens used to treat this disease should be investigated.

Although this study used the SEER database to provide considerable information on CDCK, it still presents a number of limitations. First, this study is retrospective in nature. Second, because the treatment regimen and time of chemotherapy administration are unclear, studies on the specific effects of the drug type may be limited. Finally, the lack of a centralized pathological review may lead to the misclassification of this disease. Despite these limitations, however, this study is of great benefit to clinicians seeking to assess the prognosis of CDCK and formulate appropriate treatment plans for the disease.

## Conclusions

This study used the SEER database to investigate patients with CDCK, which is a rare malignant carcinoma. Patients presenting with stage I and IV CDCK who underwent surgery had higher survival rates than patients without surgery, and patients presenting with stage IV CDCK who underwent chemotherapy had higher survival rates than patients without chemotherapy. Surgery plus chemotherapy has a survival benefit for patients presenting with stage IV compared with surgery alone or chemotherapy alone. New chemotherapy regimens should be investigated on the basis of the genetic characteristics of CDCK.

## Data Availability Statement

All raw data in this article can be obtained in the SEER program: https://seer.cancer.gov/.

## Ethics Statement

Since the data from the SEER registry were de-identified and publicly available, no institutional review board approval was necessary and no informed consent was signed for this study.

## Author Contributions

CT, XY, and WZ: study concept and design. CT and YZ: analysis data and drafting of the manuscript. SG, HL, and YZ: critical revision of the manuscript. All authors contributed to the article and approved the submitted version.

## Funding

This study was supported by the National Natural Science Foundation of China (No. 82072836).

## Conflict of Interest

The authors declare that the research was conducted in the absence of any commercial or financial relationships that could be construed as a potential conflict of interest.

## Publisher’s Note

All claims expressed in this article are solely those of the authors and do not necessarily represent those of their affiliated organizations, or those of the publisher, the editors and the reviewers. Any product that may be evaluated in this article, or claim that may be made by its manufacturer, is not guaranteed or endorsed by the publisher.

## References

[1] Gupta R Billis A Shah RB Moch H Osunkoya AO Jochum W. Carcinoma of the Collecting Ducts of Bellini and Renal Medullary Carcinoma Clinicopathologic Analysis of 52 Cases of Rare Aggressive Subtypes of Renal Cell Carcinoma With a Focus on Their Interrelationship. Am J Surg Pathol (2012) 36(9):1265–78. doi: 10.1097/PAS.0b013e3182635954 22895263

[2] Karakiewicz PI Trinh Q-D Rioux-Leclercq N de la Taille A Novara G Tostain J. Collecting Duct Renal Cell Carcinoma: A Matched Analysis of 41 Cases. Eur Urol (2007) 52(4):1140–6. doi: 10.1016/j.eururo.2007.01.070 17336449

[3] Chao D Zisman A Pantuck AJ Gitlitz BJ Freedland SJ Said JW. Collecting Duct Renal Cell Carcinoma: Clinical Study of a Rare Tumor. J Urol (2002) 167(1):71–4. doi: 10.1016/s0022-5347(05)65385-2 11743278

[4] Orsola A Trias I Raventós CX Español I Cecchini L Orsola I. Renal Collecting (Bellini) Duct Carcinoma Displays Similar Characteristics to Upper Tract Urothelial Cell Carcinoma. Urology (2005) 65(1):49–54. doi: 10.1016/j.urology.2004.08.012 15667862

[5] Pepek JM Johnstone PA Jani AB. Influence of Demographic Factors on Outcome of Collecting Duct Carcinoma: A Surveillance, Epidemiology, and End Results (SEER) Database Analysis. Clin Genitourin Cancer (2009) 7(2):E24–7. doi: 10.3816/CGC.2009.n.017 19692318

[6] Wright JL Risk MC Hotaling J Lin DW. Effect of Collecting Duct Histology on Renal Cell Cancer Outcome. J Urol (2009) 182(6):2595–9. doi: 10.1016/j.juro.2009.08.049 PMC2828767 19836761

[7] Sui W Matulay JT Robins DJ James MB Onyeji IC RoyChoudhury A. Collecting Duct Carcinoma of the Kidney: Disease Characteristics and Treatment Outcomes From the National Cancer Database. Urol Oncol (2017) 35(9):540.e13–.e18. doi: 10.1016/j.urolonc.2017.04.010 28495554

[8] Abern MR Tsivian M Polascik TJ Coogan CL. Characteristics and Outcomes of Tumors Arising From the Distal Nephron. Urology (2012) 80(1):140–6. doi: 10.1016/j.urology.2012.03.034 22626576

[9] Moch H Cubilla AL Humphrey PA Reuter VE Ulbright TM. The 2016 WHO Classification of Tumours of the Urinary System and Male Genital Organs-Part A: Renal, Penile, and Testicular Tumours. Eur Urol (2016) 70(1):93–105. doi: 10.1016/j.eururo.2016.02.029 26935559

[10] Tokuda N Naito S Matsuzaki O Nagashima Y Ozono S Igarashi T. Collecting Duct (Bellini Duct) Renal Cell Carcinoma: A Nationwide Survey in Japan. J Urol (2006) 176(1):40–3. doi: 10.1016/S0022-5347(06)00502-7 16753362

[11] Cromie W Davis C Deture F. Atypical Carcinoma of Kidney. Urology (1979) 13:315–7. doi: 10.1016/0090-4295(79)90434-5 442358

[12] Fleming S Lewi HJE. Collecting Duct Carcinoma of the Kidney. Histopathology (1986) 10(11):1131–41. doi: 10.1111/j.1365-2559.1986.tb02553.x 3542784

[13] May M Ficarra V Shariat SF Zigeuner R Chromecki T Cindolo L. Impact of Clinical and Histopathological Parameters on Disease Specific Survival in Patients With Collecting Duct Renal Cell Carcinoma: Development of a Disease Specific Risk Model. J Urol (2013) 190(2):458–63. doi: 10.1016/j.juro.2013.02.035 23434943

[14] Ciszewski S Jakimów A Smolska-Ciszewska B. Collecting (Bellini) Duct Carcinoma: A Clinical Study of a Rare Tumour and Review of the Literature. Can Urol Assoc (2015) 9(9-10):E589–93. doi: 10.5489/cuaj.2932 PMC4581923 26425219

[15] Qian X Wang Z Zhang J Wang Q Zhou P Wang S. Clinical Features and Prognostic Outcome of Renal Collecting Duct Carcinoma: 12 Cases From a Single Institution. Cancer Manage Res (2020) 12:3589–95. doi: 10.2147/cmar.S244094 PMC7245445 32547196

[16] Hasan A Abozied H Youssef A Fayad S Ismail A. A Rare Case of Collecting Duct Carcinoma With First Presentation of Respiratory Symptoms. Urol Case Rep (2020) 33:101367–. doi: 10.1016/j.eucr.2020.101367 PMC7573951 33102066

[17] Thangarasu M Raghavan D Muthu R Prabhakar V Prakash S. Collecting Duct Carcinoma of Kidney: Masquerading as Genitourinary Tuberculosis - Lessons Learnt. Urol Case Rep (2020) 29:101100. doi: 10.1016/j.eucr.2019.101100 31890601 PMC6928284

[18] Yoon SK Nam KJ Rha SH Kim JK Cho KS Kim B. Collecting Duct Carcinoma of the Kidney: CT and Pathologic Correlation. Eur J Radiol (2006) 57(3):453–60. doi: 10.1016/j.ejrad.2005.09.009 16266796

[19] Pickhardt PJ Siegal CL McLarnery JK. Collecting Duct Carcinoma of the Kidney: Are Imaging Findings Suggestive of the Diagnosis? Am J Roentgenol (2001) 176(3):627–33. doi: 10.2214/ajr.176.3.1760627 11222193

[20] Marx K Bauer J Guillou L Delaloye AB Prior J. Bellini Duct Carcinoma Visualization on F-18 FDG PET/CT. Clin Nucl Med (2009) 34(8):541–2. doi: 10.1097/RLU.0b013e3181abb768 19617742

[21] Lyu Z Liu L Li H Wang H Liu Q Chen T. Imaging Analysis of 13 Rare Cases of Renal Collecting (Bellini) Duct Carcinoma in Northern China: A Case Series and Literature Review. BMC Med Imaging (2021) 21(1):42. doi: 10.1186/s12880-021-00574-8 33676411 PMC7937320

[22] Srigley JR Delahunt B Eble JN Egevad L Epstein JI Grignon D. The International Society of Urological Pathology (ISUP) Vancouver Classification of Renal Neoplasia. Am J Surg Pathol (2013) 37(10):1469–89. doi: 10.1097/PAS.0b013e318299f2d1 24025519

[23] Fleming S. Distal Nephron Neoplasms. Semin Diagn Pathol (2015) 32(2):114–23. doi: 10.1053/j.semdp.2015.02.004 25804446

[24] Bansal P Kumar S Mittal N Kundu AK. Collecting Duct Carcinoma: A Rare Renal Tumor. Saudi J Kidney Dis Transplant (2012) 23(4):810–2. doi: 10.4103/1319-2442.98166 22805397

[25] Albadine R Schultz L Illei P Ertoy D Hicks J Sharma R. PAX8 (+)/P63 (-) Immunostaining Pattern in Renal Collecting Duct Carcinoma (CDC): A Useful Immunoprofile in the Differential Diagnosis of CDC Versus Urothelial Carcinoma of Upper Urinary Tract. Am J Surg Pathol (2010) 34(7):965–9. doi: 10.1097/PAS.0b013e3181dc5e8a PMC3505675 20463571

[26] Mennitto A Verzoni E Peverelli G Alessi A Procopio G. Management of Metastatic Collecting Duct Carcinoma: An Encouraging Result in a Patient Treated With Cabozantinib. Clin Genitourinar Cancer (2018) 16(3):E521–3. doi: 10.1016/j.clgc.2018.03.010 29656939

[27] Pagani F Colecchia M Sepe P Apollonio G Claps M Verzoni E. Collecting Ducts Carcinoma: An Orphan Disease. Literature Overview and Future Perspectives. Cancer Treat Rev (2019) 79:1–7. doi: 10.1016/j.ctrv.2019.101891 31491662

[28] Zeng Y Zhang W Li Z Zheng Y Wang Y Chen G. Personalized Neoantigen-Based Immunotherapy for Advanced Collecting Duct Carcinoma: Case Report. J ImmunoTher Cancer (2020) 8(1):e000217. doi: 10.1136/jitc-2019-000217 32439798 PMC7247377

[29] Flippot R Damarla V McGregor BA. Management of Metastatic Renal Cell Carcinoma With Variant Histologies. Urol Clinics N Am (2020) 47(3):319–27. doi: 10.1016/j.ucl.2020.04.003 32600534

[30] Ito K. Recent Advances in the Systemic Treatment of Metastatic non-Clear Cell Renal Cell Carcinomas. Int J Urol (2019) 26(9):868–77. doi: 10.1111/iju.14027 31183903

[31] Alhalabi O Karam JA Tannir NM. Evolving Role of Cytoreductive Nephrectomy in Metastatic Renal Cell Carcinoma of Variant Histology. Curr Opin Urol (2019) 29(5):521–5. doi: 10.1097/mou.0000000000000661 31305271

[32] Matsumoto H Wada T Aoki A Hoshii Y Takahashi M Aizawa S. Collecting Duct Carcinoma With Long Survival Treated by Partial Nephrectomy. Int J Urol (2001) 8(7):401–3. doi: 10.1046/j.1442-2042.2001.00321.x 11442664

[33] Kopec N Kopcza P Wrona A Salagierski M. Nephron Sparing Surgery for Metastatic Collecting Duct Carcinoma. Cent Eur J Urol (2019) 72(4):374–7. doi: 10.5173/ceju.2019.0055 PMC6979547 32015906

[34] Milowsky MI Rosmarin A Tickoo SK Papanicolaou N Nanus DM. Active Chemotherapy for Collecting Duct Carcinoma of the Kidney - A Case Report and Review of the Literature. Cancer (2002) 94(1):111–6. doi: 10.1002/cncr.10204 11815966

[35] Peyromaure M Thiounn N Scotte F Vieillefond A Debre B Oudard S. Collecting Duct Carcinoma of the Kidney: A Clinicopathological Study of 9 Cases. J Urol (2003) 170(4):1138–40. doi: 10.1097/01.ju.0000086616.40603.ad 14501710

[36] Becker F Junker K Parr M Hartmann A Füssel S Toma M. Collecting Duct Carcinomas Represent a Unique Tumor Entity Based on Genetic Alterations. PloS One (2013) 8(10):e78137. doi: 10.1371/journal.pone.0078137 24167600 PMC3805592

