# Supplementary material for: Incidence, Clinical Characteristics, and Survival of Collecting Duct Carcinoma of the Kidney: A Population-Based Study
Source: Front Oncol. 2021 Sep 14;11:727222. doi: 10.3389/fonc.2021.727222 (PMC8476990; doi:10.3389/fonc.2021.727222)
Supplement: Supplementary file 1 [file Image_1.pdf]

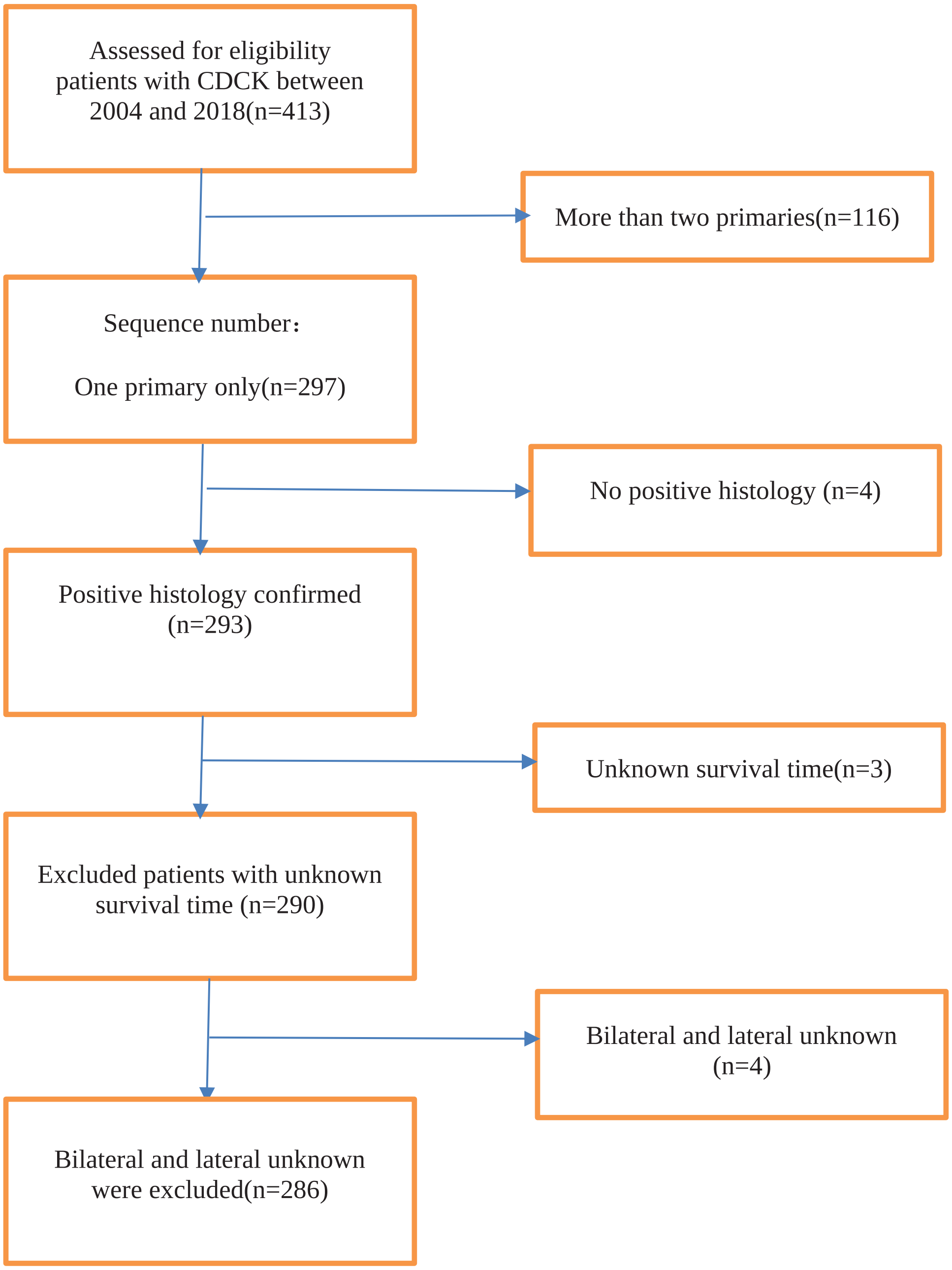

Supplementary Figure 1. Flow chart for creation of the Surveillance, Epidemiology and End Results (SEER) patient dataset.
