# Supplementary material for: Incidence, Clinical Characteristics, and Survival of Collecting Duct Carcinoma of the Kidney: A Population-Based Study
Source: Front Oncol. 2021 Sep 14;11:727222. doi: 10.3389/fonc.2021.727222 (PMC8476990; doi:10.3389/fonc.2021.727222)
Supplement: Supplementary file 2 [file Table_1.docx]

Supplementary Material

**Supplementary Table 1.**The data of chemotherapy stratified by tumor stage

| Stage | Chemotherapy | Number | Dead | Censoring |
| --- | --- | --- | --- | --- |
| Ⅰ | Yes | 0 | 0 | 0 |
|  | No | 54 | 16 | 38 |
| Ⅱ | Yes | 1 | 1 | 0 |
|  | No | 11 | 3 | 8 |
| Ⅲ | Yes | 12 | 8 | 4 |
|  | No | 57 | 29 | 28 |
| Ⅳ | Yes | 81 | 68 | 13 |
|  | No | 63 | 54 | 9 |
